# Next-generation sequencing identifies novel mitochondrial variants in pituitary adenomas

**DOI:** 10.1007/s40618-019-1005-6

**Published:** 2019-01-25

**Authors:** K. Németh, O. Darvasi, I. Likó, N. Szücs, S. Czirják, L. Reiniger, B. Szabó, P. A. Kurucz, L. Krokker, P. Igaz, A. Patócs, H. Butz

**Affiliations:** 1 2nd Department of Internal Medicine, Semmelweis University, Budapest, Hungary; 2 “Lendulet” Hereditary Endocrine Tumours Research Group, Hungarian Academy of Sciences and Semmelweis University, 46 Szentkiralyi Street, Budapest, H-1088 Hungary; 3 National Institute of Clinical Neurosciences, Budapest, Hungary; 4 1st Department of Pathology and Experimental Cancer Research, Semmelweis University, Budapest, Hungary; 5 Department of Laboratory Medicine, Semmelweis University, Budapest, Hungary; 6 Molecular Medicine Research Group, Hungarian Academy of Sciences and Semmelweis University, Budapest, Hungary

**Keywords:** Pituitary adenoma, Next-generation sequencing, Mitochondria, Genome, Genetic variations, Pathogenesis

## Abstract

**Purpose:**

Disrupted mitochondrial functions and genetic variants of mitochondrial DNA (mtDNA) have been observed in different human neoplasms. Next-generation sequencing (NGS) can be used to detect even low heteroplasmy-level mtDNA variants. We aimed to investigate the mitochondrial genome in pituitary adenomas by NGS.

**Methods:**

We analysed 11 growth hormone producing and 33 non-functioning [22 gonadotroph and 11 hormone immunonegative] pituitary adenomas using VariantPro™ Mitochondrion Panel on Illumina MiSeq instrument. Revised Cambridge Reference Sequence (rCRS) of the mtDNA was used as reference. Heteroplasmy was determined using a 3% cutoff.

**Results:**

496 variants were identified in pituitary adenomas with overall low level of heteroplasmy (7.22%). On average, 35 variants were detected per sample. Samples harbouring the highest number of variants had the highest Ki-67 indices independently of histological subtypes. We identified eight variants (A11251G, T4216C, T16126C, C15452A, T14798C, A188G, G185A, and T16093C) with different prevalences among different histological groups. T16189C was found in 40% of non-recurrent adenomas, while it was not present in the recurrent ones. T14798C and T4216C were confirmed by Sanger sequencing in all 44 samples. 100% concordance was found between NGS and Sanger method.

**Conclusions:**

NGS is a reliable method for investigating mitochondrial genome and heteroplasmy in pituitary adenomas. Out of the 496 detected variants, 414 have not been previously reported in pituitary adenoma. The high number of mtDNA variants may contribute to adenoma genesis, and some variants (i.e., T16189C) might associate with benign behaviour.

**Electronic supplementary material:**

The online version of this article (10.1007/s40618-019-1005-6) contains supplementary material, which is available to authorized users.

## Introduction

The mitochondrial genome consists of several copies of circular, double-stranded DNA molecules, covering 16,569 base pairs, 37 genes. Of these, 13 encode polypeptides of respiratory enzyme complexes, 22 encode transfer RNAs, and 2 encode ribosomal RNAs (12S, 16S) [1, 2]. Several copies of the mitochondrial DNA (mtDNA) can exist, causing co-existence of mutant and wild-type alleles in the same cell (or tissue), referred to as heteroplasmy [2]. The ratio of the mutant allele compared to the wild type is defined as degree of heteroplasmy.

Mitochondria have an essential role in apoptosis. Failure of apoptosis has been considered as a hallmark of oncogenesis. Since the first study about the mtDNA mutations in colorectal cancer has been published in 1998, extensive research has started to analyse the mitochondrial genome. The presence of frequently observed mitochondrial mutations in a variety of human neoplasms (e.g., breast, ovarian, colorectal, gastric, hepatocellular, pancreatic, prostate, lung, thyroid, renal cell cancers, and brain tumors [3]) along with the description of abnormal mitochondria in tumors raised the possibility that these mutations might have a casual role in tumor initiation and progression [4, 5].

Dasgupta et al. analysed the functional effect of the mitochondrially encoded cytochrome B (MT-CYB) gene mutation, and showed that overexpression of MT-CYB-induced tumor growth in vitro and in vivo in bladder cancer cells and increased the invasive phenotype, underlining the functional importance of this mutation [6]. Another study suggested that MT-CYB can be an immune target for CD4(+) T cells and, therefore, have implications for the immunosurveillance of mitochondrial aberrations in cancer patients [7]. In a trans-mitochondrial hybrid (cybrid) model (containing a common HeLa nucleus and mtDNA of interest), a pathogenic mitochondrially encoded ATP synthase subunit 6 (MT-ATP6) gene variant seemed to promote tumor growth by preventing apoptosis [8]. The increasing interest towards mitochondrial variants in cancer is probably due to the recognition that understanding these molecular mechanisms would take us closer to develop novel biomarkers and therapeutic strategies [9]. Still, the question whether these variants are cause or consequence of tumor progression remain unanswered.

Previous studies mostly used Sanger sequencing and MitoChip (microarray-based sequencing following PCR-amplified mitochondrial DNA) for analysing the whole mitochondrial genome. Next-generation sequencing (NGS) is a cost-effective, high-throughput, and sensitive method that has become a suitable tool to detect any DNA variants even if they are present at low level. Thus, this method is likely to be superior for identification of heteroplasmy [2, 10, 11].

Pituitary adenomas are common neoplasms accounting for 10–15% of intracranial tumors [12]. The majority (95%) occur sporadically, and despite extensive research, the molecular mechanisms of their pathogenesis are largely unknown [13]. Besides *GNAS* [guanine nucleotide-binding protein (G protein), alpha stimulating] mutations that frequently (approx. 40%) occur in somatotropinomas [14], mutations in classical tumor suppressor genes (e.g., *TP53* and *RB1*) and oncogenes (e.g., *Ras*) are rarely found in benign pituitary adenomas [15–18], suggesting the contribution of alternative mechanisms. Earlier studies identified epigenetic modifications, altered expression of cell cycle regulator cyclins and cyclin-dependent kinases (CDKs), growth factors, and their receptors, and disturbances of signal transduction pathways [18]. However, none of these changes can entirely explain the complex mechanism behind adenoma genesis.

In line with these observations, whole-exome (WES) and whole-genome sequencing (WGS) data revealed lower mutation rate in pituitary adenomas compared to other tumor types. These studies also stated that most of the variants are single-nucleotide variants (SNVs) [13, 19, 20]. Newey et al. examined non-functioning adenomas (NFPAs) by WES. They could not identify somatic mutations in genes previously reported in pituitary tumorigenesis, or associated with familial pituitary syndromes or in typical oncogenes/tumor suppressor genes. The authors also showed that there were no recurrent mutations within specific genes, suggesting that there is no common driver genes responsible for pathogenesis of NFPA [13]. Similar results were shown by another group performing WGS of growth hormone (GH) secreting pituitary adenomas [20]. Lan et al. compared invasive and non-invasive pituitary adenomas and found that the *DPCR1* (diffuse panbronchiolitis critical region 1)*, EGFL7* (EGF like domain multiple 7)*, PRDM* family (PR/SET domain family), and *LRRC50* (leucine rich repeat containing 50) can function as genetic modifiers and most likely contribute to the development of oncocytic change and invasive tumor phenotype [19].

Mitochondrial variants have only been assessed in oncocytic type of pituitary tumors so far. These studies showed high prevalence of Complex I variants which were described in association with benign behaviour [21, 22].

Here, we report for the first time data obtained using next-generation sequencing technology for analysis of the whole mitochondrial genome of pituitary tumors of different histological types, including GH-producing (GH) and clinically non-functioning [gonadotroph (GO) and hormone-immunonegative (HN)] pituitary adenomas.

## Materials and methods

### Patients

The study was approved by the Scientific and Research Committee of the Medical Research Council of Hungary (0618/15), and the samples were obtained after acquiring written informed consent from all patients.

Tissue samples were obtained from 44 patients diagnosed with pituitary adenoma, comprising 11 GH-secreting and 33 clinically non-functioning pituitary adenomas (NFPAs), including 22 gonadotroph (GO), and 11 hormone-immunonegative (HN) tumors (Table 1). Pituitary adenoma tissues were surgically removed at the National Institute of Clinical Neurosciences, Budapest, Hungary between 2015 and 2017. Histological diagnoses were performed at the 1st Department of Pathology and Experimental Cancer Research, Semmelweis University, Budapest. The clinical diagnosis of adenomas was based on patients’ hormone levels and routine histological diagnosis including immunostaining for anterior lobe hormones.

**Table 1:** Characteristics of adenoma tissue samples

Clinical diagnosis	Sex	Age	Immunhistochemistry for anterior lobe hormones	Tumor size based on preoperative MRI (mm^3^)	Ki-67 proliferation index (%)
GH producing	F	43	GH	–	1–3
GH producing	M	49	GH	–	< 1
GH producing	M	51	GH, PRL	–	< 3
GH producing	M	22	GH, PRL	11,571	3
GH producing	M	32	GH, PRL	–	3–4
GH producing	F	49	GH, PRL	2652	3–4
GH producing	F	22	GH, PRL	–	4–5
GH producing	M	35	GH, PRL	–	6
GH producing	M	30	GH, PRL	4840.495	8
GH producing	F	48	GH, PRL	–	10
GH producing	F	35	GH, PRL	–	5–6
GH producing	F	60	GH, PRL	–	< 3
NFPA	F	39	FSH	–	1–2
NFPA	M	51	FSH	15,488	2–3
NFPA	M	44	FSH	–	2–3
NFPA	F	76	FSH	3825	2–3
NFPA	M	38	FSH	–	3–4
NFPA	F	72	FSH, LH	–	1–2
NFPA	M	63	FSH, LH	41,055	< 1
NFPA	F	49	FSH, LH	3744	< 1
NFPA	M	62	FSH, LH	–	< 3
NFPA	M	67	FSH, LH	–	3–4
NFPA	M	43	FSH, LH	–	7–10
NFPA	M	64	FSH, LH	126,000	< 3
NFPA	F	74	FSH, LH	–	< 3
NFPA	F	73	FSH, LH	12,000	< 2
NFPA	F	68	FSH, LH	–	3–4
NFPA	M	73	FSH, LH	–	2–3
NFPA	F	80	FSH, LH	19,600	2–3
NFPA	F	69	FSH, LH	20,240	< 3
NFPA	M	38	FSH, LH	–	3–4
NFPA	F	37	FSH, LH	1872	7–8
NFPA	M	73	LH	13,500	2
NFPA	M	72	LH	4590	< 2
NFPA	F	43	Negative	4987.5	1
NFPA	F	50	Negative	–	2
NFPA	F	64	Negative	–	1
NFPA	F	58	Negative	3570	4
NFPA	M	73	Negative	12,144	3–4
NFPA	F	64	Negative	–	5
NFPA	M	58	Negative	35,640	5–7
NFPA	M	50	Negative	–	< 2
NFPA	F	49	Negative	–	3–4
NFPA	F	65	Negative	2523.312	3–4
NFPA	F	60	Negative	–	5

### DNA isolation

Total DNA was isolated from pituitary adenoma tissues using QIAamp Fast DNA Tissue Kit (Qiagen). DNA purity and concentration were measured using NanoDrop 1000 Spectrophotometer (Thermo Fisher Scientific). The presence of normal pituitary tissue was determined similarly to Välimäki et al. [20]. In our cohort, 43 of 44 samples contained no healthy cells, and in the remaining one tissue, the percentage of healthy pituitary cells was less than 5%.

### Next-generation sequencing for mtDNA analysis

DNA library was prepared using the VariantPro™ Mitochondrion Panel Library Preparation Kit (LC Sciences). The presence of the desired fragments and the purity of the indexed libraries were analysed on Agilent 2100 Bioanalyzer (Agilent Technologies) using High-Sensitivity DNA Analysis Kit (Agilent Technologies). The concentrations of the libraries were measured using Qubit Fluorometer (Thermo Fisher Scientific). Equimolar amounts of the 45 indexed libraries were pooled to obtain a 4 nM library mixture. After denaturing, and further diluting, the final 10 pM of library mixture was loaded into Illumina cartridge. Sequencing was performed using the Illumina MiSeq Reagent v2 kit (500 cycles) on the Illumina MiSeq instrument following the manufacturer’s instructions (Illumina).

### Bioinformatical analysis of next-generation sequencing

Sequencing data processing was performed following the Genome Analysis Toolkit (GATK) best practices guideline as follows: paired end sequencing data were exported to FASTQ file format. Reads were trimmed using Trim Galore (Babraham Bioinformatics, http://www.bioinformatics.babraham.ac.uk/projects/trim_galore/) and cutadapt [23] to remove adapters and bases, where the quality value was less than 20.

The trimmed reads were aligned with Burrows–Wheeler Aligner (BWA) [24]. Picard tools were used to sort reads (http://broadinstitute.github.io/picard). Local realignment around indels and Base Quality Score Recalibration (BQSR) were performed using GATK [25]. Variant discovery was performed in two steps: single sample variant calling was performed using MuTect2 in GATK tumor_only_mode to call all variants. This was followed by CombineVariants the single sample gVCFs (genome Variant Call Format) to the multi sample VCF (Variant Call Format). Variant effects were predicted using SnpEff [26]. Revised Cambridge Reference Sequence (rCRS) of the Human Mitochondrial DNA (NC_012920.1 gi:251831106) was applied as reference mitochondrial sequence [27]. Variants were annotated using MITOMAP mtDNA Coding Region and RNA Sequence Variants.

Reference alleles were coded as 0, variant alleles as 1 and heteroplasmy ratio was calculated by variant read number/total read for each variant and each sample. A 3% cutoff was used to identify heteroplasmy 3% as previously described [28].

### Statistical analysis

Nonparametric Kruskal–Wallis Rank test was applied for evaluating prevalence of variants among different histological groups. Multiple comparisons of mean ranks for all groups to compute post hoc comparisons of all pairs of groups were used [29]. For three groups’ comparison (with all pairs), we used the Conover–Iman test of multiple comparisons using rank sums as post hoc test in R package. We adjusted *p* values for multiple comparisons with the Benjamini and Hochberg method (BH) [30].

For comparison between two groups, the nonparametric Wilcoxon rank sum test was performed. BH was used for generation of multiple testing corrected *p* values.

For comparisons within groups, frequency of appearance of each variant (heteroplasmic or full variant number/group sample number) was also evaluated using Kruskal–Wallis Rank test and illustrated on cluster heat maps.

Proportion test was applied with “prop.test” R function which can be used for testing the null hypothesis that the proportions (probabilities of success) in several groups are the same or that they equal certain given values. This test gave similar results to Kruskal–Wallis Rank test and nonparametric Wilcoxon rank sum test.

For hierarchical cluster analysis, we used heatmap R package to draw clustered heat maps with clustering method “average” and with Euclidean distance method.

### Sanger validation of mitochondrial variants

The validation of the single-nucleotide variants (SNVs) was performed by Sanger sequencing. We sequenced the same 44 tissue samples used in NGS. To avoid co-amplification of nuclear DNA, we used a well-established method for the exclusive amplification of mitochondrial fragments as described by Ramos et al. [31, 32]. The specific mitochondrial DNA fragments were PCR-amplified and cleaned using the Clean Sweep PCR Purification Kit (A29895, Thermo Fisher Scientific). Sanger sequencing run was performed on Applied Biosystems 3130 Genetic Analyzer (Thermo Fisher Scientific) using the BigDye™ Direct Cycle Sequencing Kit (4458687, Thermo Fisher Scientific).

## Results

### Whole mitochondrial genome sequencing by NGS

Sequencing run generated an average 52,399 reads per sample (min 24,019; max 242,870) and 95 ± 1% of the reads were aligned to the mitochondrial genome. Total coverage was avg. 10,439,722 read per sample and coverage depth was 630 ± 370 (avg ± SE) reads per base.

We detected 496 single-nucleotide variants (SNVs) in all tumors. Of these, 269 variants were protein-coding of which 135 were non-synonymous and 132 were synonymous (Online Resource 1).

**Fig. 1:**
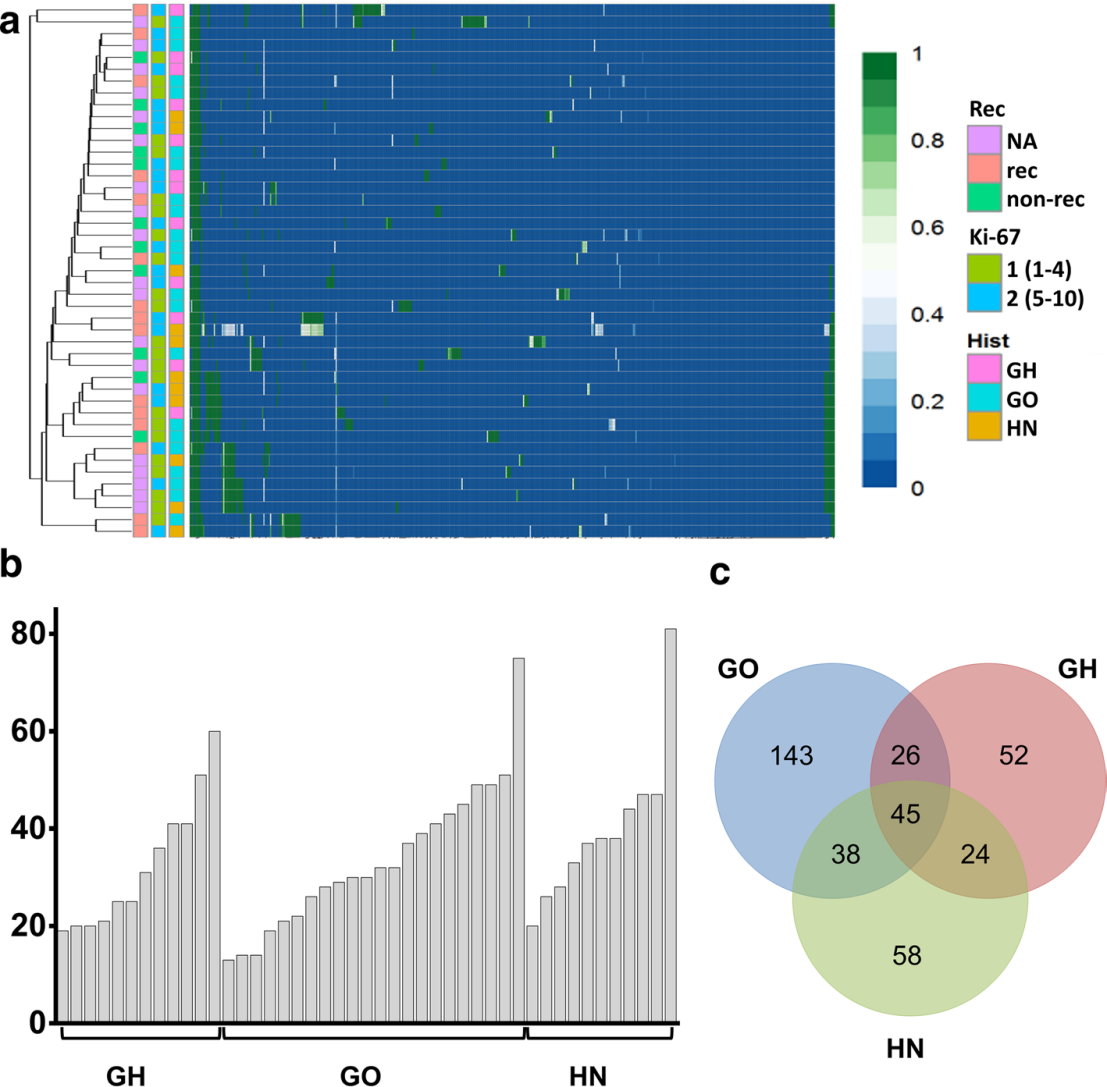
Possible associations with clinicopathological features. **a** Profile of detected variants showed overall low level of heteroplasmy and hierarchical cluster analysis could not discriminate pituitary adenoma samples based on either histological type, Ki-67 index, or recurrent/non-recurrent status. The colour scale indicates the ratio of heteroplasmy obtained by NGS, where 0 (blue) shows 100% reference allele and 1 (green) shows 100% variant allele. **b** Number of the found variants in pituitary adenoma samples, grouped by histological type and ranked from fewer to more variants. Analysing the samples harbouring the highest number of variants obtained that they have the highest Ki-67 indices independently of histological type. **c** Regarding the number of the variants, we identified 143, 58, and 52 unique variants appeared only in GO, HN, and GH-secreting adenomas, respectively. Legends: rec: recurrent, non-rec: non-recurrent, Ki-67 group 1: Ki-67 proliferation index is between 1 and 4%, Ki-67 group 2: Ki-67 proliferation index is between 5 and 10%,* Hist* histological subtype,* GH* growth hormone producing,* GO* gonadotroph,* HN* hormone immunonegative

Heteroplasmy occurred in 482 of the detected 496 variants at least in one sample and overall on low level (indicated by colours on cluster heatmap, Fig. 1a). Hormone-immunonegative (HN) adenomas showed a slightly higher heteroplasmy prevalence compared to GO and GH adenomas 8.27% vs. 6.96 and 6.72%, respectively (Table 2). This remained the same after dissection of protein-coding and non-coding variants (Table 2). In addition, we did not find difference in Ki-67 proliferation index or tumor size between sample groups harbouring low (< 50%) and high (> 50%) heteroplasmy.

**Table 2:** Prevalence of heteroplasmy of mitochondrial genome detected in pituitary adenomas

	All tumors	GO	GH	HN
All variants				
Avg (%)	7.22	6.96	6.72	8.27
SD	13.78	13.37	14.73	16.71
Protein-coding variants				
Avg (%)	6.59	6.35	6.18	7.45
SD	12.56	12.06	13.36	16.16
Non-coding variants				
Avg (%)	7.98	7.71	7.37	9.17
SD	15.14	14.79	16.24	17.34

On average, 35 variants were detected per sample. Similar results were obtained in different histological groups: we found 33, 34 and 40 variants per sample in GH-producing, gonadotroph, and hormone-immunonegative adenomas, respectively. Furthermore, samples harbouring the highest number of variants (Fig. 1b) had the highest Ki-67 indices [Ki-67: 8% (GH), 7–10% (GO), and 5% (HN)] independently of histological type.

When analysing distinct histological groups, we identified 143, 58, and 52 unique variants that appeared only in GO, HN, and GH-secreting adenomas, respectively (Fig. 1c, Online Resource 2).

Using hierarchical cluster analysis on all variants, no clear separation of the samples was identified based on histological type, Ki-67 index, or recurrent/non-recurrent status (Fig. 1a).

Nonparametric ANOVA identified eight significant variants among different histological groups. The variants: A11251G, T4216C, T16126C, C15452A, T14798C, A188G, and T16093C differentiated GH producing from HN adenomas. The prevalence of variants: T14798C, G185A, A188G, and T16093C differed between GO and HN adenomas (Table 3).

**Table 3:** Variants significantly different among different histology types of pituitary adenomas

Mitochondrial position	Region	Alleles		Gene	Prevalence (germline Caucasian)	Prevalence in our samples			Adjusted *p* value	Clinical significance
		Ref	Alt			HN (%)	GO (%)	GH (%)		
chrM_14798	Non-syn	T	C	MT-CYB	0.107	0.36	0.05	0.08	GH vs. HN: 0.0342 GO vs. HN: 0.0166	NA
chrM_4216	Non-syn	T	C	MT-ND1	NA	0.55	0.27	0.08	GH vs. HN: 0.0225	Leber’s optic atrophy
chrM_15452	Non-syn	C	A	MT-CYB	0.144	0.55	0.27	0.08	GH vs. HN: 0.0225	Neoplasm of ovary (likely pathogenic)
chrM_11251	Syn	A	G	MT-ND4	NA	0.55	0.27	0.08	GH vs. HN: 0.0225	NA
chrM_185	Non-coding	G	A	–	NA	0.27	0.00	0.08	GO vs. HN: 0.0142	NA
chrM_188	Non-coding	A	G	–	NA	0.18	0.00	0.00	GH vs. HN: 0.0246 GO vs. HN: 0.0249	NA
chrM_16093	Non-coding	T	C	–	NA	0.18	0.00	0.00	GH vs. HN: 0.0246 GO vs. HN: 0.0249	NA
chrM_16126	Non-coding	T	C	–	NA	0.55	0.27	0.08	GH vs. HN: 0.0225	NA

*MT-CYB* Mitochondrially encoded cytochrome B, *MT-ND1* mitochondrially encoded NADH:Ubiquinone Oxidoreductase Core Subunit 1, *MT-ND4* mitochondrially encoded,* NADH* Ubiquinone Oxidoreductase Core Subunit 4, *NA* not available

T16189C variant was found in 40% (6/15) of non-recurrent adenomas compared to recurrent ones, where it was not detectable (0/11) (*p* = 0.02090).

Individual variant prevalence did not show associations with Ki-67 proliferation index or tumor size.

### Technical validation

Four of the eight variants that showed differences among histological groups (G185A, A188G, T16093C, and T16126C) were localized in the D-loop region (displacement loop region, “non-coding” part of mtDNA). A11251G coded a synonymous variant, while T14798C and C15452A represented non-synonymous polymorphisms referred as rs28357681 and rs527236209, respectively, in the gene of mitochondrially encoded cytochrome B (MT-CYB). T4216C also represented a non-synonymous polymorphism as rs1599988 in mitochondrially encoded NADH:ubiquinone oxidoreductase core subunit 1 (MT-ND1). We selected rs28357681 and rs1599988 for technical validation, since these variants were protein-coding, non-synonymous variants, and these alterations showed no heteroplasmy; hence, Sanger sequencing was a reasonable option for validation. We validated the two variants on all the 44 samples and we found 100% concordance between NGS and Sanger sequencing results.

## Discussion

Mitochondrial dysfunction and mutations in mtDNA have been implicated in several human neoplasms. In this study, we analysed the whole mitochondrial genome in pituitary adenomas by NGS. The VariantPro™ Mitochondria Panel kit we used in this study has only been applied in two studies so far [33, 34]. In one of these the whole mitochondrial genome was sequenced, similarly to our study [34], while in the other, specific primers for the targeted regions were used [33]. In agreement with these studies, we observed an excellent alignment rate to mitochondrial genome and satisfactory coverage depth. This fact together with the results of validation by Sanger sequencing (100% concordance) confirmed the excellent applicability of NGS-based methods for mitochondrial genome sequencing.

Overall, we identified 496 variants in pituitary adenoma tissues compared to the human mitochondrial reference sequence. We also identified variants specific to distinct adenoma histological types. Most of the variants showed low level of heteroplasmy. This is in line with previous publications which claimed that low level of heteroplasmy was more frequent in benign tumors and occurrence of heteroplasmy increased with metastatic potential [28]. Thus, these data support our result, showing that low-level heteroplasmy is one characteristic hallmark of pituitary adenomas. In addition, samples with high and low levels of heteroplasmy did not show difference in Ki-67 proliferation index or tumor size.

Whole mitochondrial variant pattern did not show clear association with clinicopathological features. This finding is in agreement with others showing no significant associations with clinicopathological parameters including sex, age, tumor size, and duration of clinical course in tumors of the central nervous system [35]. However, when analysing the number of individual homoplasmic mitochondrial variants we found that samples harbouring the highest number of variants had the highest Ki-67 indices independent of histological type. Interestingly, increased mtDNA copy number was previously found in gliomas and the relevance of this finding needs further evaluations in pituitary adenomas [36].

We also identified eight individual variants that showed different prevalence in the different histological groups.

Four (G185A, A188G, T16093C, and T16126C) were localized in the D-loop region. Displacement loop (D-loop) region (16,024–16,569 nucleotides) is located in the mitochondrial control region (16024-576 nucleotides), often called the “non-coding” part of the mitochondrial DNA (mtDNA). The function of the D-loop is not entirely clear yet, which makes more difficult to understand the relevance of the present variants. A growing number of studies have been published about correlations between D-loop variants and clinical features [37–39]. It has been shown that D-loop mutations may play role in the pathogenesis of breast cancer [39], gastric cancer [40], bladder cancer, lung cancer, and hepatocellular carcinoma [41], while Lin et al. found that somatic mutations in the D-loop associated with a better survival in oral squamous cell carcinoma patients [42]. However, the exact functional contribution of D-loop alterations in tumorigenesis has to be further investigated [41].

Another four variants of eight that showed different prevalence in the different histological groups encoded MT-CYB, MT-ND1, and MT-ND4 (mitochondrially encoded NADH:ubiquinone oxidoreductase core subunit 4). MT-CYB is the only subunit of the Respiratory Complex III (Cytochrome bc1 complex) that is encoded in the mitochondrial genome, and it is essential for the assembly of the complex [43, 44]. Moreover, it was shown that disruption of Complex III assembly dramatically reduced the level of Complex I as well [45, 46]. T14798C has been implicated in non-muscle invasive bladder cancer [47]. The MT-ND1 gene has been examined in association with several diseases including recurrent pregnancy loss [48], colorectal cancer [49], bladder cancer [47], Parkinson’s disease [50, 51], and contradicting results have been published regarding it’s possible effect on multiple sclerosis [52] and Leber’s optic atrophy [53–55]. MT-ND4 is a subunit of the Respiratory Complex I and is needed to the assembly of the complex. Variations in ND4 sequence have been associated with macular degeneration [56], Leber’s hereditary optic neuropathy [57], mesial temporal lobe epilepsy [58], and cystic fibrosis [59]. In acute myeloid leukaemia, an association between ND4 variant and favourable prognosis has been shown [60]. In contrast, findings from other groups suggested that variations in this gene can elevate pancreatic cancer risk, or be involved in causing cisplatin resistance [61].

We found that the T16189C variant was frequent in non-recurrent adenomas, while it was absent in recurrent ones. Unfortunately, we could not analyse the primary tumor of our recurrent pituitary samples. However, a previous publication compared primary and recurrent oral squamous cell carcinomas and described that although most of the patients showed similar distribution of mutations in the index and the recurrent tumor, there were differences in the level of heteroplasmy [28].

Currently, there is no other publication about whole mtDNA analysis of different types of pituitary adenomas. A single study focused on genes encoding proteins of Respiratory Complex I in pituitary oncocytomas and another one analysed it in head and neck tumors [21, 22]. In total, 20 variants of mtDNA were identified in pituitary oncocytomas [22], and 12 others in other head and neck tumors [21]. Interestingly, none of these variants were found in more than one sample. Notably, these variants were not present in our samples emphasizing the difference between pituitary oncocytomas and pituitary adenomas of anterior lobe origin. These data are in agreement with the conclusion made by authors that unique mtDNA variants are specific for oncocytomas. Our results extend these finding by showing that unique mtDNA variants can be found in pituitary adenoma subtypes. With respect to data on pituitary tumors, the HmtDB database (http://www.hmtdb.uniba.it/) contains 19 mitochondrial variants in 19 GH ± PRL (growth hormone ± prolactin) producing adenomas. Variants described in these samples contained overlaps with our results at 82 positions including the T16189C variant (Online Resource 1). The other 414 variants identified in the current study have never been reported. However, it has to be noted that the low number of samples used in our study was a limitation of our work, and the variants identified should be further investigated on higher number of sample set.

Taken together, in this study, we reported for the first time the whole mitochondrial genome pattern of different human pituitary adenomas. We identified variants characteristic of gonadotroph, GH-producing, and hormone-immunonegative pituitary adenomas. We also observed that high number of variants may have a role in higher proliferation rate and that the T16189C variant can potentially associate with benign behaviour. Although the biological relevance of these results needs further validation, considering our data together with the low level of heteroplasmy suggest that mitochondrial genome alterations may not play a major role in the pathogenesis of pituitary adenomas. We also showed that NGS is a reliable method for the analysis of mtDNA variants with no false-positive results, and can be used for accurate quantification of even a low level of heteroplasmy.

## Electronic supplementary material

Below is the link to the electronic supplementary material.

Online Resource 1 Variants identified in pituitary adenomas. Common variants between current study and pituitary adenoma samples available in HmtDB are indicated. Current study included 44 [11 GH-producing and 33 non-functioning (gonadotroph and hormone-immunonegative)] adenomas. HmtDB samples represent only GH positive adenomas (n = 19). Out of 496 variants 414 were firstly identified by the current study (DOCX 56 kb)

Online Resource 2 Mitochondrial variants uniquely present in different adenoma types (DOCX 18 kb)
