# Supplementary material for: Next-generation sequencing identifies novel mitochondrial variants in pituitary adenomas
Source: J Endocrinol Invest. 2019 Jan 25;42(8):931–40. doi: 10.1007/s40618-019-1005-6 (PMC6647476; doi:10.1007/s40618-019-1005-6)
Supplement: Supplementary file 1 — Online Resource 1 Variants identified in pituitary adenomas. Common variants between current study and pituitary adenoma samples available in HmtDB are indicated. Current study included 44 [11 GH-producing and 33 non-functioning (gonadotroph and hormone-immunonegative)] adenomas. HmtDB samples represent only GH positive adenomas (n = 19). Out of 496 variants 414 were firstly identified by the current study (DOCX 56 kb) [file 40618_2019_1005_MOESM1_ESM.docx]

**Supplementary Table 1** Variants identified in pituitary adenomas

| **Position** | **Type/gene** | **Current study (n=44)** | | | | **Variants present in HmtDB (GH±PRL adenoma, n=19)** | | |
| --- | --- | --- | --- | --- | --- | --- | --- | --- |
|  |  | **Ref** | **Var** | **Number of samples** | **Firstly identified variants in PA** | **Identified nucleotide change** | **Number of samples** |  |
| chrM_28 | D-loop/NA | A | C | 6 | novel |  |  |  |
| chrM_72 | D-loop/NA | T | C | 2 | novel |  |  |  |
| chrM_73 | D-loop/NA | A | G | 22 |  | G-A | 10 |  |
| chrM_146 | D-loop/NA | T | C | 2 |  | C-T | 16 |  |
| chrM_150 | D-loop/NA | C | T | 3 |  | C-T | 2 |  |
| chrM_152 | D-loop/NA | T | C | 9 |  | C-T | 14 |  |
| chrM_183 | D-loop/NA | A | G | 1 | novel |  |  |  |
| chrM_185 | D-loop/NA | G | A | 4 | novel |  |  |  |
| chrM_188 | D-loop/NA | A | G | 2 | novel |  |  |  |
| chrM_189 | D-loop/NA | A | G | 1 | novel |  |  |  |
| chrM_195 | D-loop/NA | T | C | 8 |  | C-T | 18 |  |
| chrM_199 | D-loop/NA | T | C | 1 | novel |  |  |  |
| chrM_204 | D-loop/NA | T | C | 2 |  | T-C | 1 |  |
| chrM_207 | D-loop/NA | G | A | 2 | novel |  |  |  |
| chrM_228 | D-loop/NA | G | A | 5 | novel |  |  |  |
| chrM_235 | D-loop/NA | A | G | 1 | novel |  |  |  |
| chrM_239 | D-loop/NA | T | C | 1 | novel |  |  |  |
| chrM_247 | D-loop/NA | GA | G | 1 |  | A-G | 19 |  |
| chrM_263 | D-loop/NA | A | G | 43 | novel |  |  |  |
| chrM_295 | D-loop/NA | C | T | 5 |  | C-T | 3 |  |
| chrM_302 | D-loop/NA | A | AC | 3 | novel |  |  |  |
| chrM_310 | D-loop/NA | T | TC | 38 |  | T-C | 1 |  |
| chrM_316 | D-loop/NA | G | A,C | 2 |  | G-C | 1 |  |
| chrM_317 | D-loop/NA | CTT | C | 1 | novel |  |  |  |
| chrM_320 | D-loop/NA | CTGG | C | 1 | novel |  |  |  |
| chrM_368 | D-loop/NA | A | G | 1 | novel |  |  |  |
| chrM_374 | D-loop/NA | A | G | 1 | novel |  |  |  |
| chrM_385 | D-loop/NA | A | G | 1 | novel |  |  |  |
| chrM_456 | D-loop/NA | C | T | 1 | novel |  |  |  |
| chrM_462 | D-loop/NA | C | T | 6 |  | C-T | 3 |  |
| chrM_489 | D-loop/NA | T | C | 8 |  | T-N | 1 |  |
| chrM_497 | D-loop/NA | C | T | 1 |  | C-T | 2 |  |
| chrM_499 | D-loop/NA | G | A | 2 |  | G-N | 1 |  |
| chrM_513 | D-loop/NA | GCA | G | 8 |  | G-N | 1 |  |
| chrM_545 | D-loop/NA | G | C | 2 |  | G-N | 1 |  |
| chrM_564 | D-loop/NA | G | A | 1 |  | G-N | 1 |  |
| chrM_593 | tRNA/TRNF | T | TC | 1 |  | T-N | 1 |  |
| chrM_709 | rRNA/RNR1 | G | A | 8 |  | G-A | 1 |  |
| chrM_750 | rRNA/RNR1 | A | G | 43 | novel |  |  |  |
| chrM_866 | rRNA/RNR1 | A | G | 1 | novel |  |  |  |
| chrM_877 | rRNA/RNR1 | G | A | 1 | novel |  |  |  |
| chrM_879 | rRNA/RNR1 | T | C | 2 | novel |  |  |  |
| chrM_930 | rRNA/RNR1 | G | A | 3 | novel |  |  |  |
| chrM_933 | rRNA/RNR1 | G | A | 1 | novel |  |  |  |
| chrM_951 | rRNA/RNR1 | G | A | 1 | novel |  |  |  |
| chrM_955 | rRNA/RNR1 | A | AC | 3 | novel |  |  |  |
| chrM_961 | rRNA/RNR1 | T | G | 1 | novel |  |  |  |
| chrM_989 | rRNA/RNR1 | T | G | 1 | novel |  |  |  |
| chrM_992 | rRNA/RNR1 | TA | T | 3 | novel |  |  |  |
| chrM_1030 | rRNA/RNR1 | G | A | 1 | novel |  |  |  |
| chrM_1189 | rRNA/RNR1 | T | C | 1 |  | T-C | 2 |  |
| chrM_1438 | rRNA/RNR1 | A | G | 43 | novel |  |  |  |
| chrM_1465 | rRNA/RNR1 | C | G | 4 | novel |  |  |  |
| chrM_1591 | rRNA/RNR1 | C | A | 1 | novel |  |  |  |
| chrM_1718 | rRNA/RNR2 | A | AG | 1 | novel |  |  |  |
| chrM_1719 | rRNA/RNR2 | G | A | 2 | novel |  |  |  |
| chrM_1811 | rRNA/RNR2 | A | G | 3 |  | A-G | 2 |  |
| chrM_1824 | rRNA/RNR2 | T | C | 1 | novel |  |  |  |
| chrM_1888 | rRNA/RNR2 | G | A | 7 | novel |  |  |  |
| chrM_1893 | rRNA/RNR2 | A | C | 1 | novel |  |  |  |
| chrM_1958 | rRNA/RNR2 | G | A | 1 | novel |  |  |  |
| chrM_2129 | STS/RNR2 | GA | G | 26 | novel |  |  |  |
| chrM_2135 | STS/RNR2 | AC | A | 1 | novel |  |  |  |
| chrM_2149 | STS/RNR2 | G | A | 1 | novel |  |  |  |
| chrM_2203 | STS/RNR2 | G | A | 1 | novel |  |  |  |
| chrM_2245 | rRNA/RNR2 | A | G | 1 | novel |  |  |  |
| chrM_2387 | rRNA/RNR2 | T | C | 1 | novel |  |  |  |
| chrM_2392 | rRNA/RNR2 | T | C | 1 |  | T-Y | 1 |  |
| chrM_2407 | rRNA/RNR2 | T | C | 1 | novel |  |  |  |
| chrM_2416 | rRNA/RNR2 | T | C | 1 | novel |  |  |  |
| chrM_2487 | rRNA/RNR2 | A | C | 18 | novel |  |  |  |
| chrM_2523 | rRNA/RNR2 | C | A | 1 | novel |  |  |  |
| chrM_2525 | rRNA/RNR2 | C | A | 13 | novel |  |  |  |
| chrM_2623 | rRNA/RNR2 | A | G | 1 | novel |  |  |  |
| chrM_2702 | rRNA/RNR2 | G | A | 1 | novel |  |  |  |
| chrM_2706 | rRNA/RNR2 | A | G | 18 |  | G-A | 19 |  |
| chrM_2772 | rRNA/RNR2 | C | T | 1 | novel |  |  |  |
| chrM_2807 | rRNA/RNR2 | T | G | 0 | novel |  |  |  |
| chrM_2817 | rRNA/RNR2 | G | A | 1 | novel |  |  |  |
| chrM_2850 | rRNA/RNR2 | T | C | 1 | novel |  |  |  |
| chrM_3010 | rRNA/RNR2 | G | A | 11 |  | G-A | 4 |  |
| chrM_3127 | rRNA/RNR2 | G | A | 1 | novel |  |  |  |
| chrM_3197 | rRNA/RNR2 | T | C | 3 |  | T-C | 2 |  |
| chrM_3240 | tRNA/TRNL1 | C | G | 2 | novel |  |  |  |
| chrM_3264 | tRNA/TRNL1 | T | C | 1 | novel |  |  |  |
| chrM_3314 | CDS/ND1 | T | A,C | 7 | novel |  |  |  |
| chrM_3317 | CDS/ND1 | CCA | C | 6 | novel |  |  |  |
| chrM_3380 | CDS/ND1 | G | A | 2 | novel |  |  |  |
| chrM_3392 | CDS/ND1 | G | A | 2 | novel |  |  |  |
| chrM_3414 | CDS/ND1 | C | G | 9 | novel |  |  |  |
| chrM_3480 | CDS/ND1 | A | G | 1 |  | A-G | 2 |  |
| chrM_3492 | CDS/ND1 | A | C | 5 | novel |  |  |  |
| chrM_3511 | CDS/ND1 | A | C | 2 | novel |  |  |  |
| chrM_3552 | CDS/ND1 | T | A | 1 | novel |  |  |  |
| chrM_3565 | CDS/ND1 | A | AC | 2 | novel |  |  |  |
| chrM_3604 | CDS/ND1 | CTAG | C | 7 | novel |  |  |  |
| chrM_3670 | CDS/ND1 | G | A | 1 | novel |  |  |  |
| chrM_3748 | CDS/ND1 | AT | A | 1 | novel |  |  |  |
| chrM_3796 | CDS/ND1 | A | G | 2 | novel |  |  |  |
| chrM_3832 | CDS/ND1 | C | T | 1 | novel |  |  |  |
| chrM_3915 | CDS/ND1 | G | A | 1 | novel |  |  |  |
| chrM_3918 | CDS/ND1 | G | A | 1 | novel |  |  |  |
| chrM_3997 | CDS/ND1 | A | AC | 1 | novel |  |  |  |
| chrM_4000 | CDS/ND1 | A | AG | 1 | novel |  |  |  |
| chrM_4002 | CDS/ND1 | TATAATA | T | 1 | novel |  |  |  |
| chrM_4048 | CDS/ND1 | G | A | 1 | novel |  |  |  |
| chrM_4071 | CDS/ND1 | C | T | 1 | novel |  |  |  |
| chrM_4086 | CDS/ND1 | C | T | 1 | novel |  |  |  |
| chrM_4122 | CDS/ND1 | A | G | 1 | novel |  |  |  |
| chrM_4155 | CDS/ND1 | C | A | 2 |  | C-T | 1 |  |
| chrM_4164 | CDS/ND1 | A | G | 1 | novel |  |  |  |
| chrM_4216 | CDS/ND1 | T | C | 13 |  | T-C | 4 |  |
| chrM_4227 | CDS/ND1 | A | G | 1 | novel |  |  |  |
| chrM_4401 | STS/NA | A | G | 1 | novel |  |  |  |
| chrM_4435 | tRNA/TRNM | A | G | 1 | novel |  |  |  |
| chrM_4558 | CDS/ND2 | G | A | 1 | novel |  |  |  |
| chrM_4580 | CDS/ND2 | G | A | 2 | novel |  |  |  |
| chrM_4639 | CDS/ND2 | T | C | 1 | novel |  |  |  |
| chrM_4646 | CDS/ND2 | T | C | 2 | novel |  |  |  |
| chrM_4715 | CDS/ND2 | A | G | 1 | novel |  |  |  |
| chrM_4720 | CDS/ND2 | G | A | 1 | novel |  |  |  |
| chrM_4727 | CDS/ND2 | A | G | 1 | novel |  |  |  |
| chrM_4755 | CDS/ND2 | T | C | 1 | novel |  |  |  |
| chrM_4769 | CDS/ND2 | A | G | 42 | novel |  |  |  |
| chrM_4793 | CDS/ND2 | A | G | 1 | novel |  |  |  |
| chrM_4796 | CDS/ND2 | C | T | 1 |  | C-T | 1 |  |
| chrM_4820 | CDS/ND2 | G | A | 1 | novel |  |  |  |
| chrM_4917 | CDS/ND2 | A | G | 7 | novel |  |  |  |
| chrM_4937 | CDS/ND2 | T | C | 1 | novel |  |  |  |
| chrM_4966 | CDS/ND2 | GT | G | 2 | novel |  |  |  |
| chrM_4974 | CDS/ND2 | G | A | 1 | novel |  |  |  |
| chrM_5031 | CDS/ND2 | G | A | 1 | novel |  |  |  |
| chrM_5060 | CDS/ND2 | C | A | 3 | novel |  |  |  |
| chrM_5147 | CDS/ND2 | G | A | 3 | novel |  |  |  |
| chrM_5186 | CDS/ND2 | A | G | 2 | novel |  |  |  |
| chrM_5198 | CDS/ND2 | A | G | 2 | novel |  |  |  |
| chrM_5228 | CDS/ND2 | C | G | 3 | novel |  |  |  |
| chrM_5262 | CDS/ND2 | G | A | 1 | novel |  |  |  |
| chrM_5263 | CDS/ND2 | C | T | 1 | novel |  |  |  |
| chrM_5293 | CDS/ND2 | G | A | 1 | novel |  |  |  |
| chrM_5325 | CDS/ND2 | A | G | 1 | novel |  |  |  |
| chrM_5334 | CDS/ND2 | A | G | 1 | novel |  |  |  |
| chrM_5351 | CDS/ND2 | A | G | 1 | novel |  |  |  |
| chrM_5460 | CDS/ND2 | G | A | 1 |  | G-A | 2 |  |
| chrM_5471 | CDS/ND2 | G | A | 2 | novel |  |  |  |
| chrM_5530 | tRNA/TRNW | C | A | 1 | novel |  |  |  |
| chrM_5687 | tRNA/TRNN | CT | C | 6 | novel |  |  |  |
| chrM_5691 | tRNA/TRNN | GT | G | 4 | novel |  |  |  |
| chrM_5693 | tRNA/TRNN | TA | T | 2 | novel |  |  |  |
| chrM_5703 | tRNA/TRNN | G | A | 1 | novel |  |  |  |
| chrM_5801 | tRNA/TRNC | T | C | 1 | novel |  |  |  |
| chrM_5906 | CDS/COX1 | G | A | 1 | novel |  |  |  |
| chrM_5999 | CDS/COX1 | T | C | 2 | novel |  |  |  |
| chrM_6047 | CDS/COX1 | A | G | 2 | novel |  |  |  |
| chrM_6050 | CDS/COX1 | T | C | 1 | novel |  |  |  |
| chrM_6100 | CDS/COX1 | T | C | 1 | novel |  |  |  |
| chrM_6126 | CDS/COX1 | A | G | 1 | novel |  |  |  |
| chrM_6221 | CDS/COX1 | T | C | 2 | novel |  |  |  |
| chrM_6227 | CDS/COX1 | T | C | 1 | novel |  |  |  |
| chrM_6371 | STS/COX1 | C | T | 2 | novel |  |  |  |
| chrM_6386 | STS/COX1 | C | T | 1 | novel |  |  |  |
| chrM_6425 | STS/COX1 | T | C | 1 | novel |  |  |  |
| chrM_6455 | STS/COX1 | C | T | 1 | novel |  |  |  |
| chrM_6464 | STS/COX1 | C | A | 1 | novel |  |  |  |
| chrM_6554 | STS/COX1 | C | T | 1 | novel |  |  |  |
| chrM_6556 | STS/COX1 | C | A | 0 | novel |  |  |  |
| chrM_6626 | STS/COX1 | T | C | 2 | novel |  |  |  |
| chrM_6680 | STS/COX1 | T | C | 1 | novel |  |  |  |
| chrM_6741 | STS/COX1 | AT | A | 1 | novel |  |  |  |
| chrM_6756 | STS/COX1 | T | C | 1 | novel |  |  |  |
| chrM_6768 | STS/COX1 | G | A | 1 | novel |  |  |  |
| chrM_6776 | STS/COX1 | T | C | 1 | novel |  |  |  |
| chrM_6779 | STS/COX1 | A | G | 1 | novel |  |  |  |
| chrM_6791 | STS/COX1 | A | G | 2 | novel |  |  |  |
| chrM_6845 | STS/COX1 | C | T | 7 | novel |  |  |  |
| chrM_6851 | STS/COX1 | C | T | 1 | novel |  |  |  |
| chrM_7022 | CDS/COX1 | T | C | 1 | novel |  |  |  |
| chrM_7024 | CDS/COX1 | T | C | 1 | novel |  |  |  |
| chrM_7028 | CDS/COX1 | C | T | 24 |  | T-C | 11 |  |
| chrM_7080 | CDS/COX1 | T | C | 1 |  | T-C | 1 |  |
| chrM_7162 | STS/NA | G | A | 1 | novel |  |  |  |
| chrM_7196 | STS/NA | C | A | 1 | novel |  |  |  |
| chrM_7270 | STS/NA | T | C | 1 | novel |  |  |  |
| chrM_7299 | STS/NA | A | G | 1 | novel |  |  |  |
| chrM_7300 | STS/NA | T | G | 0 | novel |  |  |  |
| chrM_7303 | STS/NA | T | G | 0 | novel |  |  |  |
| chrM_7312 | STS/NA | T | G | 0 | novel |  |  |  |
| chrM_7315 | STS/NA | T | G | 0 | novel |  |  |  |
| chrM_7559 | STS/NA | A | G | 1 | novel |  |  |  |
| chrM_7601 | STS/NA | CA | C | 23 | novel |  |  |  |
| chrM_7603 | STS/NA | AG | A | 4 | novel |  |  |  |
| chrM_7604 | STS/NA | GT | G | 3 | novel |  |  |  |
| chrM_7621 | STS/NA | T | C | 1 | novel |  |  |  |
| chrM_7645 | STS/NA | T | C | 1 | novel |  |  |  |
| chrM_7684 | STS/NA | T | C | 1 | novel |  |  |  |
| chrM_7706 | STS/NA | GC | G | 1 | novel |  |  |  |
| chrM_7707 | STS/NA | C | G | 18 | novel |  |  |  |
| chrM_7709 | STS/NA | CT | C | 5 | novel |  |  |  |
| chrM_7714 | STS/NA | C | T | 1 | novel |  |  |  |
| chrM_7789 | STS/NA | G | A | 1 | novel |  |  |  |
| chrM_7819 | STS/NA | C | G | 1 | novel |  |  |  |
| chrM_7853 | STS/NA | G | A | 1 | novel |  |  |  |
| chrM_7879 | STS/NA | AT | A | 1 | novel |  |  |  |
| chrM_7910 | STS/NA | G | A | 1 | novel |  |  |  |
| chrM_7963 | STS/NA | A | G | 1 | novel |  |  |  |
| chrM_8014 | STS/NA | A | T | 1 | novel |  |  |  |
| chrM_8020 | STS/NA | G | A | 1 | novel |  |  |  |
| chrM_8026 | STS/NA | A | T | 1 | novel |  |  |  |
| chrM_8153 | STS/NA | G | A | 1 | novel |  |  |  |
| chrM_8155 | STS/NA | G | A | 2 | novel |  |  |  |
| chrM_8270 | STS/NA | C | CACCCCCTCT | 1 | novel |  |  |  |
| chrM_8277 | STS/NA | T | C | 1 | novel |  |  |  |
| chrM_8278 | STS/NA | CTA | C | 1 | novel |  |  |  |
| chrM_8279 | STS/NA | T | C | 1 | novel |  |  |  |
| chrM_8280 | STS/NA | AC | A | 1 | novel |  |  |  |
| chrM_8281 | STS/NA | C | A | 18 | novel |  |  |  |
| chrM_8286 | STS/NA | T | C | 1 | novel |  |  |  |
| chrM_8287 | STS/NA | CTAGAG | C | 1 |  | C-Y | 1 |  |
| chrM_8308 | STS/NA | A | G | 1 | novel |  |  |  |
| chrM_8334 | STS/NA | G | C | 1 | novel |  |  |  |
| chrM_8348 | STS/NA | A | G | 2 | novel |  |  |  |
| chrM_8387 | STS/NA | G | A | 1 | novel |  |  |  |
| chrM_8431 | STS/NA | C | T | 1 | novel |  |  |  |
| chrM_8440 | STS/NA | A | G | 1 | novel |  |  |  |
| chrM_8448 | STS/NA | T | C | 1 | novel |  |  |  |
| chrM_8473 | STS/NA | T | C | 1 |  | T-C | 1 |  |
| chrM_8482 | STS/NA | A | C | 4 | novel |  |  |  |
| chrM_8483 | STS/NA | A | T | 4 | novel |  |  |  |
| chrM_8503 | STS/NA | T | C | 2 | novel |  |  |  |
| chrM_8521 | STS/NA | A | G | 1 | novel |  |  |  |
| chrM_8584 | CDS/ATP6 | G | A | 1 | novel |  |  |  |
| chrM_8596 | CDS/ATP6 | A | G | 1 | novel |  |  |  |
| chrM_8697 | CDS/ATP6 | G | A | 7 | novel |  |  |  |
| chrM_8701 | CDS/ATP6 | A | G | 2 |  | G-A | 19 |  |
| chrM_8786 | CDS/ATP6 | TC | T | 2 | novel |  |  |  |
| chrM_8788 | CDS/ATP6 | CT | C | 2 | novel |  |  |  |
| chrM_8789 | CDS/ATP6 | T | C | 1 | novel |  |  |  |
| chrM_8818 | CDS/ATP6 | C | T | 2 | novel |  |  |  |
| chrM_8860 | CDS/ATP6 | A | G | 42 | novel |  |  |  |
| chrM_8869 | CDS/ATP6 | A | G | 1 | novel |  |  |  |
| chrM_8926 | CDS/ATP6 | C | T | 1 | novel |  |  |  |
| chrM_8950 | CDS/ATP6 | G | A | 1 | novel |  |  |  |
| chrM_9033 | CDS/ATP6 | A | G | 1 | novel |  |  |  |
| chrM_9044 | CDS/ATP6 | T | C | 0 | novel |  |  |  |
| chrM_9055 | CDS/ATP6 | G | A | 0 |  | G-A | 2 |  |
| chrM_9110 | CDS/ATP6 | T | C | 1 | novel |  |  |  |
| chrM_9128 | CDS/ATP6 | T | C | 1 | novel |  |  |  |
| chrM_9156 | CDS/ATP6 | A | G | 1 | novel |  |  |  |
| chrM_9182 | CDS/ATP6 | G | A | 1 | novel |  |  |  |
| chrM_9380 | STS/COX3 | G | A | 1 | novel |  |  |  |
| chrM_9398 | STS/COX3 | A | G | 1 | novel |  |  |  |
| chrM_9445 | CDS/COX3 | GA | G | 1 | novel |  |  |  |
| chrM_9477 | CDS/COX3 | G | A | 2 |  | G-A | 2 |  |
| chrM_9492 | CDS/COX3 | G | A | 1 | novel |  |  |  |
| chrM_9520 | CDS/COX3 | G | A | 1 | novel |  |  |  |
| chrM_9527 | CDS/COX3 | C | T | 1 | novel |  |  |  |
| chrM_9540 | CDS/COX3 | T | C | 2 |  | C-T | 19 |  |
| chrM_9545 | CDS/COX3 | A | G | 1 | novel |  |  |  |
| chrM_9548 | CDS/COX3 | G | A | 1 | novel |  |  |  |
| chrM_9554 | CDS/COX3 | G | A | 1 | novel |  |  |  |
| chrM_9575 | CDS/COX3 | G | A | 1 | novel |  |  |  |
| chrM_9698 | CDS/COX3 | T | C | 1 |  | T-C | 2 |  |
| chrM_9819 | CDS/COX3 | G | A | 1 | novel |  |  |  |
| chrM_9824 | CDS/COX3 | T | C | 1 | novel |  |  |  |
| chrM_9899 | CDS/COX3 | T | C | 1 | novel |  |  |  |
| chrM_9949 | CDS/COX3 | T | C | 1 | novel |  |  |  |
| chrM_9966 | CDS/COX3 | G | A | 1 |  | G-A | 1 |  |
| chrM_10014 | tRNA/TRNG | G | A | 1 | novel |  |  |  |
| chrM_10084 | CDS/ND3 | T | C | 2 | novel |  |  |  |
| chrM_10162 | CDS/ND3 | C | A | 8 | novel |  |  |  |
| chrM_10177 | CDS/ND3 | G | A | 1 | novel |  |  |  |
| chrM_10192 | CDS/ND3 | C | T | 1 | novel |  |  |  |
| chrM_10217 | CDS/ND3 | A | G | 1 | novel |  |  |  |
| chrM_10253 | CDS/ND3 | T | C | 1 | novel |  |  |  |
| chrM_10316 | CDS/ND3 | AAT | A | 1 | novel |  |  |  |
| chrM_10334 | CDS/ND3 | CT | C | 5 | novel |  |  |  |
| chrM_10334 | CDS/ND3 | C | T | 1 | novel |  |  |  |
| chrM_10337 | CDS/ND3 | AT | A | 1 | novel |  |  |  |
| chrM_10345 | CDS/ND3 | T | C | 1 | novel |  |  |  |
| chrM_10360 | CDS/ND3 | G | A | 1 | novel |  |  |  |
| chrM_10394 | CDS/ND3 | C | T | 1 | novel |  |  |  |
| chrM_10398 | CDS/ND3 | A | G | 9 |  | G-A | 19 |  |
| chrM_10400 | CDS/ND3 | C | T | 2 | novel |  |  |  |
| chrM_10463 | tRNA/TRNR | T | C | 7 | novel |  |  |  |
| chrM_10550 | CDS/ND4L | A | G | 1 |  | A-G | 2 |  |
| chrM_10630 | CDS/ND4L | T | C | 1 | novel |  |  |  |
| chrM_10813 | CDS/ND4 | CAA | C | 1 | novel |  |  |  |
| chrM_10873 | CDS/ND4 | T | C | 2 |  | C-T | 19 |  |
| chrM_10946 | CDS/ND4 | A | AC | 1 | novel |  |  |  |
| chrM_10948 | CDS/ND4 | C | T | 1 | novel |  |  |  |
| chrM_11009 | CDS/ND4 | T | C | 1 | novel |  |  |  |
| chrM_11016 | CDS/ND4 | G | A | 1 | novel |  |  |  |
| chrM_11031 | CDS/ND4 | GA | G | 2 | novel |  |  |  |
| chrM_11150 | CDS/ND4 | G | A | 1 | novel |  |  |  |
| chrM_11168 | CDS/ND4 | G | T | 1 | novel |  |  |  |
| chrM_11169 | CDS/ND4 | G | A | 1 | novel |  |  |  |
| chrM_11203 | CDS/ND4 | C | T | 1 | novel |  |  |  |
| chrM_11251 | CDS/ND4 | A | G | 13 |  | A-G | 3 |  |
| chrM_11253 | CDS/ND4 | T | C | 1 | novel |  |  |  |
| chrM_11299 | CDS/ND4 | T | C | 1 |  | T-C | 2 |  |
| chrM_11307 | CDS/ND4 | C | G | 2 | novel |  |  |  |
| chrM_11332 | CDS/ND4 | C | T | 2 | novel |  |  |  |
| chrM_11337 | CDS/ND4 | A | G | 1 | novel |  |  |  |
| chrM_11377 | CDS/ND4 | G | A | 1 | novel |  |  |  |
| chrM_11402 | CDS/ND4 | T | C | 1 | novel |  |  |  |
| chrM_11423 | CDS/ND4 | G | A | 1 | novel |  |  |  |
| chrM_11465 | CDS/ND4 | T | C | 2 | novel |  |  |  |
| chrM_11467 | CDS/ND4 | A | G | 5 |  | A-G | 4 |  |
| chrM_11652 | CDS/ND4 | TA | T | 2 | novel |  |  |  |
| chrM_11655 | CDS/ND4 | CA | C | 0 | novel |  |  |  |
| chrM_11656 | CDS/ND4 | AG | A | 1 | novel |  |  |  |
| chrM_11719 | CDS/ND4 | G | A | 21 |  | A-G | 10 |  |
| chrM_11812 | CDS/ND4 | A | G | 4 | novel |  |  |  |
| chrM_11832 | CDS/ND4 | G | A | 1 | novel |  |  |  |
| chrM_11866 | CDS/ND4 | AC | A | 1 | novel |  |  |  |
| chrM_11878 | CDS/ND4 | T | C | 2 | novel |  |  |  |
| chrM_11914 | CDS/ND4 | G | A | 1 |  | A-G | 19 |  |
| chrM_11959 | CDS/ND4 | A | G | 1 | novel |  |  |  |
| chrM_12103 | CDS/ND4 | C | A | 1 | novel |  |  |  |
| chrM_12127 | CDS/ND4 | G | A | 1 | novel |  |  |  |
| chrM_12161 | tRNA/TRNH | T | C | 1 | novel |  |  |  |
| chrM_12308 | tRNA/TRNL2 | A | G | 5 |  | A-G | 5 |  |
| chrM_12372 | CDS/ND5 | G | A | 5 |  | G-A | 5 |  |
| chrM_12373 | CDS/ND5 | A | G | 1 | novel |  |  |  |
| chrM_12390 | CDS/ND5 | C | T | 1 | novel |  |  |  |
| chrM_12405 | CDS/ND5 | C | T | 1 | novel |  |  |  |
| chrM_12417 | CDS/ND5 | CA | C,CAA | 4 | novel |  |  |  |
| chrM_12458 | CDS/ND5 | CA | C | 2 | novel |  |  |  |
| chrM_12543 | CDS/ND5 | CACAACCCAA | C | 1 | novel |  |  |  |
| chrM_12596 | CDS/ND5 | TA | T | 2 | novel |  |  |  |
| chrM_12612 | CDS/ND5 | A | G | 7 |  | A-G | 3 |  |
| chrM_12633 | CDS/ND5 | C | A | 3 | novel |  |  |  |
| chrM_12634 | CDS/ND5 | A | G | 1 |  | A-G | 1 |  |
| chrM_12645 | CDS/ND5 | C | T | 1 | novel |  |  |  |
| chrM_12684 | CDS/ND5 | G | A | 7 | novel |  |  |  |
| chrM_12705 | CDS/ND5 | C | T | 10 |  | T-C | 18 |  |
| chrM_12716 | CDS/ND5 | C | T | 1 | novel |  |  |  |
| chrM_12764 | CDS/ND5 | G | A | 1 | novel |  |  |  |
| chrM_12773 | CDS/ND5 | G | A | 1 | novel |  |  |  |
| chrM_12810 | CDS/ND5 | A | G | 1 | novel |  |  |  |
| chrM_12811 | CDS/ND5 | T | C | 2 | novel |  |  |  |
| chrM_12937 | CDS/ND5 | A | G | 2 | novel |  |  |  |
| chrM_12957 | CDS/ND5 | T | C | 1 | novel |  |  |  |
| chrM_13036 | CDS/ND5 | C | T | 1 | novel |  |  |  |
| chrM_13062 | CDS/ND5 | A | G | 3 | novel |  |  |  |
| chrM_13063 | CDS/ND5 | G | A | 1 | novel |  |  |  |
| chrM_13095 | CDS/ND5 | T | C | 6 | novel |  |  |  |
| chrM_13102 | CDS/ND5 | G | A | 1 | novel |  |  |  |
| chrM_13105 | CDS/ND5 | A | G | 6 |  | G-A | 19 |  |
| chrM_13117 | CDS/ND5 | A | G | 1 | novel |  |  |  |
| chrM_13158 | CDS/ND5 | A | G | 2 | novel |  |  |  |
| chrM_13169 | CDS/ND5 | T | C | 1 | novel |  |  |  |
| chrM_13230 | CDS/ND5 | CA | C | 1 | novel |  |  |  |
| chrM_13263 | CDS/ND5 | A | G | 1 | novel |  |  |  |
| chrM_13271 | CDS/ND5 | T | C | 1 | novel |  |  |  |
| chrM_13304 | CDS/ND5 | AC | A | 2 | novel |  |  |  |
| chrM_13336 | CDS/ND5 | T | C | 1 | novel |  |  |  |
| chrM_13368 | CDS/ND5 | G | A | 7 | novel |  |  |  |
| chrM_13385 | CDS/ND5 | T | C | 1 | novel |  |  |  |
| chrM_13437 | CDS/ND5 | T | A | 14 | novel |  |  |  |
| chrM_13441 | CDS/ND5 | A | C | 25 | novel |  |  |  |
| chrM_13528 | CDS/ND5 | A | G | 1 | novel |  |  |  |
| chrM_13563 | CDS/ND5 | AT | A | 8 | novel |  |  |  |
| chrM_13565 | CDS/ND5 | CT | C | 1 | novel |  |  |  |
| chrM_13567 | CDS/ND5 | AT | A | 8 | novel |  |  |  |
| chrM_13570 | CDS/ND5 | AC | A | 6 | novel |  |  |  |
| chrM_13613 | CDS/ND5 | T | C | 1 | novel |  |  |  |
| chrM_13617 | CDS/ND5 | T | C | 2 |  | T-C | 2 |  |
| chrM_13681 | CDS/ND5 | A | G | 1 | novel |  |  |  |
| chrM_13708 | CDS/ND5 | G | A | 8 |  | G-A | 4 |  |
| chrM_13759 | CDS/ND5 | G | A | 1 |  | G-A | 1 |  |
| chrM_13788 | CDS/ND5 | C | A | 1 | novel |  |  |  |
| chrM_13823 | CDS/ND5 | T | C | 1 | novel |  |  |  |
| chrM_13824 | CDS/ND5 | A | G | 1 | novel |  |  |  |
| chrM_13825 | CDS/ND5 | G | A | 1 | novel |  |  |  |
| chrM_13834 | CDS/ND5 | A | G | 1 | novel |  |  |  |
| chrM_13846 | CDS/ND5 | C | A | 1 | novel |  |  |  |
| chrM_13857 | CDS/ND5 | A | G | 1 | novel |  |  |  |
| chrM_13965 | CDS/ND5 | T | C | 1 | novel |  |  |  |
| chrM_13966 | CDS/ND5 | A | G | 2 | novel |  |  |  |
| chrM_13984 | CDS/ND5 | CT | C | 1 | novel |  |  |  |
| chrM_14003 | CDS/ND5 | C | T | 1 | novel |  |  |  |
| chrM_14016 | CDS/ND5 | G | A | 1 | novel |  |  |  |
| chrM_14110 | CDS/ND5 | T | C | 2 | novel |  |  |  |
| chrM_14167 | CDS/ND6 | C | T | 1 |  | C-T | 2 |  |
| chrM_14233 | CDS/ND6 | A | G | 4 | novel |  |  |  |
| chrM_14318 | CDS/ND6 | T | C | 1 | novel |  |  |  |
| chrM_14319 | CDS/ND6 | T | C | 1 | novel |  |  |  |
| chrM_14344 | CDS/ND6 | A | G | 1 | novel |  |  |  |
| chrM_14346 | CDS/ND6 | C | A | 1 | novel |  |  |  |
| chrM_14435 | CDS/ND6 | T | C | 1 | novel |  |  |  |
| chrM_14470 | CDS/ND6 | T | C | 2 | novel |  |  |  |
| chrM_14587 | CDS/ND6 | AC | A | 1 | novel |  |  |  |
| chrM_14588 | CDS/ND6 | C | A | 1 | novel |  |  |  |
| chrM_14620 | CDS/ND6 | C | T | 2 | novel |  |  |  |
| chrM_14687 | tRNA/TRNE | A | G | 1 | novel |  |  |  |
| chrM_14766 | CDS/CYTB | C | T | 22 |  | T-C | 19 |  |
| chrM_14783 | CDS/CYTB | T | C | 2 | novel |  |  |  |
| chrM_14793 | CDS/CYTB | A | G | 2 | novel |  |  |  |
| chrM_14798 | CDS/CYTB | T | C | 6 |  | T-C | 3 |  |
| chrM_14893 | CDS/CYTB | A | G | 1 | novel |  |  |  |
| chrM_14905 | CDS/CYTB | G | A | 7 | novel |  |  |  |
| chrM_14925 | CDS/CYTB | CA | C | 10 | novel |  |  |  |
| chrM_14927 | CDS/CYTB | AC | A | 26 | novel |  |  |  |
| chrM_14929 | CDS/CYTB | C | CTAG | 5 | novel |  |  |  |
| chrM_14971 | CDS/CYTB | T | C | 1 | novel |  |  |  |
| chrM_15043 | CDS/CYTB | G | A | 3 | novel |  |  |  |
| chrM_15050 | CDS/CYTB | CT | C | 2 | novel |  |  |  |
| chrM_15052 | CDS/CYTB | AT | A | 2 | novel |  |  |  |
| chrM_15054 | CDS/CYTB | AT | A | 1 | novel |  |  |  |
| chrM_15062 | CDS/CYTB | T | C | 1 | novel |  |  |  |
| chrM_15172 | CDS/CYTB | G | A | 1 | novel |  |  |  |
| chrM_15204 | CDS/CYTB | TC | T | 0 | novel |  |  |  |
| chrM_15218 | CDS/CYTB | A | G | 2 |  | A-G | 1 |  |
| chrM_15245 | CDS/CYTB | G | A | 1 | novel |  |  |  |
| chrM_15249 | CDS/CYTB | A | G | 1 | novel |  |  |  |
| chrM_15299 | CDS/CYTB | T | C | 1 | novel |  |  |  |
| chrM_15301 | CDS/CYTB | G | A | 2 | novel |  |  |  |
| chrM_15314 | CDS/CYTB | G | A | 1 | novel |  |  |  |
| chrM_15322 | CDS/CYTB | A | G | 1 | novel |  |  |  |
| chrM_15323 | CDS/CYTB | G | A | 1 | novel |  |  |  |
| chrM_15326 | CDS/CYTB | A | G | 43 | novel |  |  |  |
| chrM_15331 | CDS/CYTB | C | T | 1 | novel |  |  |  |
| chrM_15343 | CDS/CYTB | CT | C | 1 | novel |  |  |  |
| chrM_15452 | CDS/CYTB | C | A | 13 |  | C-A | 3 |  |
| chrM_15457 | CDS/CYTB | C | T | 1 | novel |  |  |  |
| chrM_15487 | CDS/CYTB | A | T | 1 | novel |  |  |  |
| chrM_15607 | CDS/CYTB | A | G | 7 | novel |  |  |  |
| chrM_15620 | CDS/CYTB | CT | C | 3 | novel |  |  |  |
| chrM_15693 | CDS/CYTB | T | C | 2 | novel |  |  |  |
| chrM_15700 | CDS/CYTB | C | T | 1 | novel |  |  |  |
| chrM_15732 | CDS/CYTB | C | G | 1 | novel |  |  |  |
| chrM_15734 | CDS/CYTB | G | A | 2 | novel |  |  |  |
| chrM_15750 | CDS/CYTB | T | C | 1 | novel |  |  |  |
| chrM_15764 | CDS/CYTB | G | A | 1 | novel |  |  |  |
| chrM_15873 | CDS/CYTB | T | C | 1 | novel |  |  |  |
| chrM_15904 | tRNA/TRNT | C | T | 2 | novel |  |  |  |
| chrM_15927 | tRNA/TRNT | G | A | 1 | novel |  |  |  |
| chrM_15928 | tRNA/TRNT | G | A | 7 | novel |  |  |  |
| chrM_16036 | D-loop/NA | G | A | 1 | novel |  |  |  |
| chrM_16067 | D-loop/NA | C | T | 1 | novel |  |  |  |
| chrM_16069 | D-loop/NA | C | T | 6 |  | C-T | 4 |  |
| chrM_16086 | D-loop/NA | T | C | 1 | novel |  |  |  |
| chrM_16092 | D-loop/NA | T | C | 1 | novel |  |  |  |
| chrM_16093 | D-loop/NA | T | C | 2 |  | T-C | 1 |  |
| chrM_16126 | D-loop/NA | T | C | 13 |  | T-C | 4 |  |
| chrM_16129 | D-loop/NA | G | A | 5 |  | A-G | 19 |  |
| chrM_16134 | D-loop/NA | C | T | 2 | novel |  |  |  |
| chrM_16148 | D-loop/NA | C | T | 1 | novel |  |  |  |
| chrM_16162 | D-loop/NA | A | G | 1 | novel |  |  |  |
| chrM_16163 | D-loop/NA | A | G | 3 | novel |  |  |  |
| chrM_16164 | D-loop/NA | A | G | 0 | novel |  |  |  |
| chrM_16182 | D-loop/NA | A | AC | 1 | novel |  |  |  |
| chrM_16183 | D-loop/NA | A | C | 2 |  | A-C | 1 |  |
| chrM_16185 | D-loop/NA | C | CT | 1 | novel |  |  |  |
| chrM_16186 | D-loop/NA | C | T | 2 | novel |  |  |  |
| chrM_16189 | D-loop/NA | T | C | 9 |  | C-T | 19 |  |
| chrM_16192 | D-loop/NA | C | T | 3 |  | C-T | 1 |  |
| chrM_16193 | D-loop/NA | C | T | 1 |  | C-T | 1 |  |
| chrM_16194 | D-loop/NA | A | G | 1 | novel |  |  |  |
| chrM_16209 | D-loop/NA | T | C | 1 | novel |  |  |  |
| chrM_16221 | D-loop/NA | C | T | 1 | novel |  |  |  |
| chrM_16223 | D-loop/NA | C | T | 4 |  | T-C | 14 |  |
| chrM_16224 | D-loop/NA | T | C | 1 |  | T-C | 2 |  |
| chrM_16234 | D-loop/NA | C | T | 2 | novel |  |  |  |
| chrM_16239 | D-loop/NA | C | T | 1 |  | C-T | 1 |  |
| chrM_16240 | D-loop/NA | A | G | 1 | novel |  |  |  |
| chrM_16256 | D-loop/NA | C | T | 1 |  | C-T | 1 |  |
| chrM_16261 | D-loop/NA | C | T | 2 |  | C-T | 3 |  |
| chrM_16263 | D-loop/NA | T | C | 1 | novel |  |  |  |
| chrM_16265 | D-loop/NA | A | T | 1 | novel |  |  |  |
| chrM_16266 | D-loop/NA | C | T | 1 | novel |  |  |  |
| chrM_16270 | D-loop/NA | C | T | 3 |  | C-T | 2 |  |
| chrM_16278 | D-loop/NA | C | T | 2 |  | T-C | 19 |  |
| chrM_16287 | D-loop/NA | C | T | 1 | novel |  |  |  |
| chrM_16288 | D-loop/NA | T | C | 1 | novel |  |  |  |
| chrM_16293 | D-loop/NA | A | G | 0 | novel |  |  |  |
| chrM_16294 | D-loop/NA | C | T | 8 | novel |  |  |  |
| chrM_16296 | D-loop/NA | C | T | 3 | novel |  |  |  |
| chrM_16297 | D-loop/NA | T | C | 1 | novel |  |  |  |
| chrM_16298 | D-loop/NA | T | C | 4 | novel |  |  |  |
| chrM_16302 | D-loop/NA | A | G | 1 | novel |  |  |  |
| chrM_16304 | D-loop/NA | T | C | 4 |  | T-C | 1 |  |
| chrM_16311 | D-loop/NA | T | C | 4 |  | C-T | 19 |  |
| chrM_16327 | D-loop/NA | C | T | 1 | novel |  |  |  |
| chrM_16354 | D-loop/NA | C | T | 1 | novel |  |  |  |
| chrM_16355 | D-loop/NA | C | T | 1 | novel |  |  |  |
| chrM_16356 | D-loop/NA | T | C | 5 |  | T-C | 1 |  |
| chrM_16362 | D-loop/NA | T | C | 3 |  | T-C | 1 |  |
| chrM_16366 | D-loop/NA | C | T | 1 | novel |  |  |  |
| chrM_16399 | D-loop/NA | A | G | 1 | novel |  |  |  |
| chrM_16482 | D-loop/NA | A | G | 1 | novel |  |  |  |
| chrM_16519 | D-loop/NA | T | C | 31 |  | C-T | 19 |  |
| chrM_16526 | D-loop/NA | G | A | 1 | novel |  |  |  |
| chrM_16527 | D-loop/NA | C | T | 1 | novel |  |  |  |
| chrM_16545 | D-loop/NA | T | C | 2 | novel |  |  |  |
