# Supplementary material for: Next-generation sequencing identifies novel mitochondrial variants in pituitary adenomas
Source: J Endocrinol Invest. 2019 Jan 25;42(8):931–40. doi: 10.1007/s40618-019-1005-6 (PMC6647476; doi:10.1007/s40618-019-1005-6)
Supplement: Supplementary file 2 — Online Resource 2 Mitochondrial variants uniquely present in different adenoma types (DOCX 18 kb) [file 40618_2019_1005_MOESM2_ESM.docx]

**Supplementary Table 2** Mitochondrial variants uniquely present in different adenoma types.

| **Gonadotroph adenoma (143)** | | **Growth hormone- secreting adenoma (52)** | **Hormone-immunonegative adenoma (58)** |
| --- | --- | --- | --- |
| chrM_183 | chrM_9899 | chrM_199 | chrM_188 |
| chrM_235 | chrM_9949 | chrM_368 | chrM_189 |
| chrM_239 | chrM_9966 | chrM_374 | chrM_497 |
| chrM_247 | chrM_10217 | chrM_456 | chrM_866 |
| chrM_317 | chrM_10253 | chrM_951 | chrM_1189 |
| chrM_320 | chrM_10946 | chrM_2245 | chrM_1958 |
| chrM_385 | chrM_11009 | chrM_2387 | chrM_2416 |
| chrM_564 | chrM_11016 | chrM_2407 | chrM_2850 |
| chrM_593 | chrM_11031 | chrM_2772 | chrM_3480 |
| chrM_877 | chrM_11169 | chrM_3670 | chrM_3832 |
| chrM_879 | chrM_11203 | chrM_4048 | chrM_3918 |
| chrM_933 | chrM_11253 | chrM_4071 | chrM_4435 |
| chrM_961 | chrM_11377 | chrM_4164 | chrM_4755 |
| chrM_1030 | chrM_11402 | chrM_4639 | chrM_5801 |
| chrM_1718 | chrM_11866 | chrM_5263 | chrM_5906 |
| chrM_1824 | chrM_11914 | chrM_5325 | chrM_6227 |
| chrM_1893 | chrM_11959 | chrM_5351 | chrM_6425 |
| chrM_2392 | chrM_12103 | chrM_5460 | chrM_6851 |
| chrM_3264 | chrM_12127 | chrM_5703 | chrM_7022 |
| chrM_3380 | chrM_12390 | chrM_6386 | chrM_7559 |
| chrM_3414 | chrM_12634 | chrM_6455 | chrM_7621 |
| chrM_3552 | chrM_12645 | chrM_6680 | chrM_7910 |
| chrM_3565 | chrM_12684 | chrM_6768 | chrM_8020 |
| chrM_3915 | chrM_12716 | chrM_6776 | chrM_8270 |
| chrM_4086 | chrM_12764 | chrM_7080 | chrM_8308 |
| chrM_4122 | chrM_12773 | chrM_7299 | chrM_8440 |
| chrM_4227 | chrM_12957 | chrM_7684 | chrM_8596 |
| chrM_4401 | chrM_13036 | chrM_7819 | chrM_9128 |
| chrM_4558 | chrM_13063 | chrM_7853 | chrM_9520 |
| chrM_4715 | chrM_13102 | chrM_8153 | chrM_9698 |
| chrM_4727 | chrM_13169 | chrM_8869 | chrM_10334 |
| chrM_4793 | chrM_13263 | chrM_8926 | chrM_10360 |
| chrM_4796 | chrM_13613 | chrM_9110 | chrM_10394 |
| chrM_4937 | chrM_13681 | chrM_9548 | chrM_10550 |
| chrM_4974 | chrM_13759 | chrM_9575 | chrM_10630 |
| chrM_5031 | chrM_13788 | chrM_9824 | chrM_10813 |
| chrM_5262 | chrM_13823 | chrM_10192 | chrM_10948 |
| chrM_5293 | chrM_13825 | chrM_10345 | chrM_11299 |
| chrM_5334 | chrM_13834 | chrM_12161 | chrM_11337 |
| chrM_6126 | chrM_13984 | chrM_12373 | chrM_11832 |
| chrM_6464 | chrM_14003 | chrM_12405 | chrM_12810 |
| chrM_6554 | chrM_14318 | chrM_12811 | chrM_13117 |
| chrM_6756 | chrM_14344 | chrM_13528 | chrM_13230 |
| chrM_6779 | chrM_14893 | chrM_13824 | chrM_13336 |
| chrM_7024 | chrM_14971 | chrM_14435 | chrM_13857 |
| chrM_7196 | chrM_15172 | chrM_15323 | chrM_13965 |
| chrM_7270 | chrM_15218 | chrM_16182 | chrM_14167 |
| chrM_7645 | chrM_15245 | chrM_16256 | chrM_14319 |
| chrM_7714 | chrM_15249 | chrM_16287 | chrM_14687 |
| chrM_7789 | chrM_15322 | chrM_16297 | chrM_15062 |
| chrM_7963 | chrM_15487 | chrM_16526 | chrM_15331 |
| chrM_8014 | chrM_15734 | chrM_16527 | chrM_16086 |
| chrM_8026 | chrM_15750 |  | chrM_16093 |
| chrM_8277 | chrM_15927 |  | chrM_16185 |
| chrM_8279 | chrM_16067 |  | chrM_16224 |
| chrM_8281 | chrM_16092 |  | chrM_16239 |
| chrM_8431 | chrM_16148 |  | chrM_16240 |
| chrM_8448 | chrM_16162 |  | chrM_16366 |
| chrM_8473 | chrM_16193 |  |  |
| chrM_8521 | chrM_16194 |  |  |
| chrM_8584 | chrM_16209 |  |  |
| chrM_9033 | chrM_16221 |  |  |
| chrM_9156 | chrM_16263 |  |  |
| chrM_9182 | chrM_16265 |  |  |
| chrM_9380 | chrM_16266 |  |  |
| chrM_9398 | chrM_16288 |  |  |
| chrM_9492 | chrM_16302 |  |  |
| chrM_9527 | chrM_16327 |  |  |
| chrM_9545 | chrM_16355 |  |  |
| chrM_9554 | chrM_16399 |  |  |
| chrM_9819 | chrM_16482 |  |  |
|  | chrM_16545 |  |  |
